# Analogues of Artificial Human Box C/D Small Nucleolar RNA As Regulators of Alternative Splicing  of a pre-mRNA Target

**Published:** 2012

**Authors:** G.A. Stepanov, D.V. Semenov, E.V. Kuligina, O.A. Koval, I.V. Rabinov, Y.Y. Kit, V.A. Richter

**Affiliations:** Institute of Chemical Biology and Fundamental Medicine, Siberian Branch, Russian Academy of Sciences; Institute of Cell Biology, National Academy of Sciences of Ukraine

**Keywords:** small nucleolar box C/D RNAs, post-transcriptional RNA modification, alternative splicing of pre-mRNA

## Abstract

Small nucleolar RNAs (snoRNAs) play a key role in ribosomal RNA (rRNA) biogenesis. Box C/D snoRNAs guide the site-specific 2’-O-ribose methylation of nucleotides in rRNAs and small nuclear RNAs (snRNAs). A number of box C/D snoRNAs and their fragments have recently been reported to regulate post-transcriptional modifications and the alternative splicing of pre-mRNA. Artificial analogues of U24 snoRNAs directed to nucleotides in 28S and 18S rRNAs, as well as pre-mRNAs and mature mRNAs of human heat shock cognate protein (hsc70), were designed and synthesized in this study. It was found that after the transfection of MCF-7 human cells with artificial box C/D RNAs in complex with lipofectamine, snoRNA analogues penetrated into cells and accumulated in the cytoplasm and nucleus. It was demonstrated that the transfection of cultured human cells with artificial box C/D snoRNA targeted to pre-mRNAs induce partial splicing impairments. It was found that transfection with artificial snoRNAs directed to 18S and 28S rRNA nucleotides, significant for ribosome functioning, induce a decrease in MCF-7 cell viability*.*

## INTRODUCTION

The class of small nucleolar RNAs (snoRNAs) consists of two major families: box C/D RNAs and box H/ACA RNAs. These RNAs act as a recognizing and targeting element in RNA–ribonucleoprotein complexes and participate in the modification of nucleotides in eukaryotic ribosomal RNAs. The RNAs belonging to the box C/D RNA family guide the 2’-O-methylation of rRNA nucleotides. Box C/D RNAs contain conserved structural elements, CUGA (box D) and RUGAUGA (box C), near the 5’ and 3’ termini, respectively. The box C/D RNAs possess a guide sequence region (a sequence complementary to the region of an RNA target). Certain RNAs contain two guide sequences and two box C/D pairs (C, D, C’, and D’) [1].

*Cavaille J. et al.* [2] demonstrated that if the RNA contains structural elements determining its membership in the family of box C/D RNAs, then it is sufficient to have the corresponding region of the box C/D snoRNA (complementary to the RNA target) in order to determine the methylation target. It was shown that 2’-O-methylation of RNA nucleotides without natural 2’-O-methyl groups can be guided by analogues of box C/D RNAs [2].

One of the key approaches to the study of the properties of box C/D RNAs is the design of DNA constructs that are expressed in a cell yielding either short non-natural snoRNAs or pre-mRNA fragments, the processing of which results in the formation of snoRNAs targeted to the pre-specified rRNA nucleotides [2]. This approach was used to design methods for the directed nucleotide modification in eukaryotic RNAs and for mapping functionally important rRNA sites, which are sensitive to *de* *novo* 2’-O-methylation [3, 4]. The range of targets for artificial small nucleolar RNAs is not confined to rRNAs and snRNAs. The participation of snoRNAs in mRNA maturation is of increasing interest. It has already been revealed that box C/D RNAs can interact both with the products of transcription by RNA-polymerase I localized in the nucleolus and with RNA-polymerase II products [2]. Moreover, small nucleolar RNA HBII-52 (MBII-52) takes part in the processing of pre-mRNA of the serotonin receptor 5-HT _2C_ R [5, 6]. Thus, the structure of box C/D small nucleolar RNAs is a promising basis for the development of constructs for the directed regulation of gene expression in human cells.

The effect of synthetic analogues of natural box C/D RNAs on pre-mRNA-target splicing and processing of 18S and 28S rRNA in human cells was investigated in this study. The analogues of human U24 box C/D RNA directed to pre-mRNA of human heat shock cognate protein hsc70 and human rRNA were designed. The transfection of the MCF-7 cell line (human breast adenocarcinoma cells) with synthetic analogues was shown to result in a partial disruption of splicing (the elimination of an exon from the pre-mRNA-target). It was found that the transfection of MCF-7 cells with synthetic analogues of box C/D RNAs directed to rRNAs induces a decrease in cell viability.

## EXPERIMENTAL

**Artificial box C/D RNAs synthesis**

Synthetic analogues of box C/D RNAs were obtained via the *in* *vitro* transcription of PCR-amplified DNA templates with T7 RNA polymerase (Fermentas, Lithuania).

**Transfection of MCF-7 cells with synthetic RNAs. Isolation of total cellular RNA**

MCF-7 cells (from the Russian cell culture collection of vertebrates, Institute of Cytology, Russian Academy of Sciences, St. Petersburg) were cultured in an IMDM medium with 10 mM L-glutamine and 40 µg/ml of gentamicin in the presence of 10% fetal bovine serum at 37 ^о^ С. Synthetic analogues of box C/D RNAs were pre-incubated with the lipofectamine reagent (Invitrogen, United States) according to the manufacturer’s protocol and were subsequently added to the culture medium. After the MCF-7 cells had been incubated for 18 h, total RNA was isolated using the Trizol reagent (Invitrogen, United States) according to the manufacturer’s protocol.

**Isolation of cytoplasmic and nuclear fractions of MCF-7 cell lysate**

Upon completion of incubation, the MCF-7 cells were ice-cooled. The medium was collected. The cells were washed twice with phosphate buffered saline (PBS) and lysed on ice for 10 min (0.5% Triton X-100 in buffer A containing 150 mM NaCl, 50 mM Tris-HCl pH 7.5, 10 mM EDTA). The lysate was suspended and coated onto a 10% sucrose solution in buffer A, followed by centrifugation for 20 min at 600 *g* . The isolation of RNA from the supernatant (the cytoplasmic fraction of MCF-7) and nucleolar precipitate suspended in buffer A was carried out using the Trizol reagent. RNA concentrations in the samples were determined spectrophotometrically (λ = 260 nm), taking into account the extinction coefficient for RNA (ε _260_  = 25 l/mol cm). 

**Analysis of pre-mRNA **

*HSPA8* **splicing variants by RT-PCR**

The reverse transcription of pre-mRNA in the *HSPA8 * and cDNA amplification was performed in the reaction mixture for RT-PCR “Real Best Master Mix RT” (Vektor-Best, Novosibirsk, Russia) using the primers hsp2.1 (5’-ACTGAACGGTTGATCGGTGA-3’) and hsp8.2 (5’-AGATGAGCACGTTTCTTTCT-3’). The products were analyzed in a 4% polyacrylamide gel. The quantity of amplification products in the gel was ascertained using the Gel-Pro Analyzer 3.1 software. Sanger sequencing was performed using fluorescently labelled terminators of DNA-polymerase in the BigDye 3.1 mixture, followed by the separation of DNA on an ABI3100 analyzer (Applied Biosystems, Inter-Institute Sequencing Centre, Siberian Branch of the Russian Academy of Sciences).

**Fluorescently labelled box C/D RNA synthesis and analysis of the accumulation of fluorescently labelled RNA in human cells**

Fluorescently labelled RNA was obtained via *in* *vitro* transcription by T7 RNA polymerase (Fermentas, Lithuania) using Flu-12-UTP (Biosan, Novosibirsk, Russia). The RNA transcript was isolated via ion-pair RP-HPLC on a Milichrome A-02 liquid chromatograph using the ProntoSIL-120-5-C18 sorbent and a 2.0 × 7.5 mm column. The accumulation of fluorescently labelled RNA in MCF-7 cells was analyzed via fluorescence microscopy (The Centre for Collective Use of Microscopic Analysis of Biological Objects, Siberian Branch of the Russian Academy of Science). For this purpose, 3 × 10 ^4^ MCF-7 cells were seeded onto a slide plate of the Culture Slide chamber (BD Falcon, United States), then they were transfected with fluorescently labelled RNA after 24 h, followed by incubation for 18 h. The medium was removed post-incubation. The cells were washed twice with PBS; the specimens were embedded into a drop of the DAPI/Antifade dye (Millipore, United States) and covered with a cover slip. The specimens were analyzed on an Axioskop 2 Plus microscope (Carl Zeiss, Germany).

**Analysis of 2’-O-methylation of G1702 of the 18S rRNA**

**Fig. 1:**
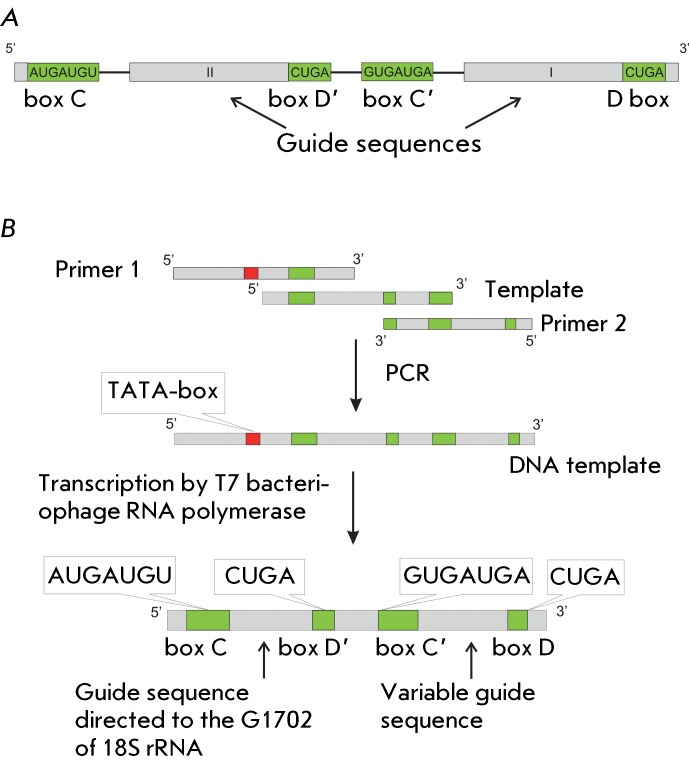
(A) – Structure of U24 box C/D RNA;  (B) – Scheme of synthesis of the artificial box C/D RNA.

2’-O-methyl groups of rRNA were detected via partial alkaline hydrolysis as previously described in [7]. The total RNA of the MCF-7 cells (2.5–5.0 µg) was incubated in a 50 mM Na _2_ CO _3_ solution (pH 9.0) for 18 min at 90 ^о^ С. The hydrolysis products were recovered by ethanol precipitation. Reverse transcription was carried out using primer 18.1702 (5’-GCCGATCCGAGGGCCTCACT-3’), complementary to the region 1731–1750 of the 18S rRNA, using M-MLV reverse transcriptase (Biosan, Novosibirsk, Russia). The rRNA region was sequenced via reverse transcription in the presence of ddNTP, according to [8].

**Analysis of MCF-7 cell viability via the MTT assay**

In order to analyze the effect of the analogues of box C/D RNAs on the viability of MCF-7 cells, the cells were cultured in a 96-well plate (3 × 10 ^4 ^ cells per well). After 24 h, the RNA–lipofectamine complex was added to the culture mixture until concentrations of 3.0, 10.0, and 70.0 nM were obtained. MCF-7 cells were incubated with RNA for 3 days; the MTT solution in PBS was subsequently added to the medium until a final concentration of 0.5 mg/ml. The mixture was incubated at 37°С for 90 min. After the medium was removed, MTT-formazan crystals were dissolved in 100 µl of isopropanol. The absorbance of the solution was determined (λ = 570 nm, control at λ = 620 nm) on an Apollo 8 LB 912 multichannel spectrophotometer (Berthold Technologies, Germany). The data were presented as a decrease in viability (100% – MTT index) against the control (cells incubated under identical conditions with lipofectamine alone).

## RESULTS

The effect of the synthetic analogues of box C/D RNAs on RNA processing in human cells was studied using the designed analogues of human natural U24 box C/D RNA. Human U24 RNA contains CUGA and AUGAUGU (GUGAUGA) sequences (for D and C (C’) boxes, respectively) and two guide sequences directing the 2’-O-methylation of C2338 and C2352 in 28S rRNA ( *Fig* . * 1A) * [9]. The resulting analogues contain conserved regions that are identical to U24 box C/D RNA and the regions complementary to those of the RNA-targets designed so that the target nucleotide in the RNA target was complementary to the fifth nucleotide upstream from box D (D’) (CUGA) of the analogue of box C/D RNA [2]. All the resulting RNAs contained two sets of box C/D RNA (C/D and C’/D’, respectively) and thus two guide sequences ( *Fig. 1B* ).

One of the guide sequences (the D-box-dependent one) was directed to pre-mRNA nucleotides of the *HSPA8* gene encoding the human heat shock cognate protein (hsc70). Suppressing the expression of hsp-70-related proteins, including the hsc70 protein, results in the death of cultured cancer cells. The *HSPA8* gene is considered to be a promising target for gene-targeted cancer therapy [10]. Nucleotides, the modification of which may have a negative effect on the excision of the second intron upon pre-mRNA splicing, were selected for application as targets of the synthetic analogues: adenosine – the splicing branch point, splice donor and splice acceptor sites, the first and the last intron nucleotides. The second guide sequence (the D’-dependent sequence) was directed to G1702 in human 18S rRNA. The sequences of the analogues of box C/D RNAs are specified in *Table 1* .

It was ascertained via the transfection of MCF-7 cells with synthetic analogues of box C/D RNA and analysis of the variants of the alternative splicing of pre-mRNA of the *HSPA8 * gene that both variants of alternative splicing of this pre-mRNA (the major and minor ones) can be identified in the control MCF-7 cells ( *Fig* . * 2, lane K* ). It was demonstrated via the sequencing of these forms that they differ by either the presence or absence of the second exon. The content of the minor form, i.e., the form without the second exon, increases in the cells transfected with synthetic box C/D RNAs directed to the nucleotides that are key ones for pre-mRNA splicing *(Fig* . *2A, lanes 1–5* ). Thus, it was found that the analogues of box C/D RNAs directed to the selected pre-mRNA nucleotides of the *HSPA8* gene have an impact on the pre-mRNA target splicing and result in the excision of the second exon.

**Table 1:** Synthetic analogues of U24 box C/D RNA directed to the key nucleotides involved in the splicing of pre-mRNA of the gene HSPA8

Notation	Nucleotide sequence*	Target nucleotide in the hsc70 pre-mRNA
РМ.7	5’-UGCAGAUGAUGUAAAAUAGCGACGGGCGGUGCUGAGAG AUGGUGAUGACAAAUGAAAACACUUUCAAUCUGAUGCA-3’	Adenosine – splicing branch point
РМ.8	5’-UGCAGAUGAUGUAAAAUAGCGACGGGCGGUGCUGAGAG AUGGUGAUGAAAAUUAGGAACUCACCAAAACUGAUGCA-3’	Splice donor site
РМ.9	5’-UGCAGAUGAUGUAAAAUAGCGACGGGCGGUGCUGAGAG AUGGUGAUGAAAAUUAGGAACUCACCAAACUGAUGCA-3’	First intron nucleotide
РМ.10	5’-UGCAGAUGAUGUAAAAUAGCGACGGGCGGUGCUGAGAG AUGGUGAUGAACAGAUGCCAAACGUCUGAUCUGAUGCA-3’	Splice acceptor site
РМ.11	5’-UGCAGAUGAUGUAAAAUAGCGACGGGCGGUGCUGAGAG AUGGUGAUGAUACAGAUGCCAAACGUCUGACUGAUGCA-3’	Last intron nucleotide

*AUGAUGU – conserved elements of box C/D RNA; A – nucleotides that are complementary to the target nucleotide.

**Fig. 2:**
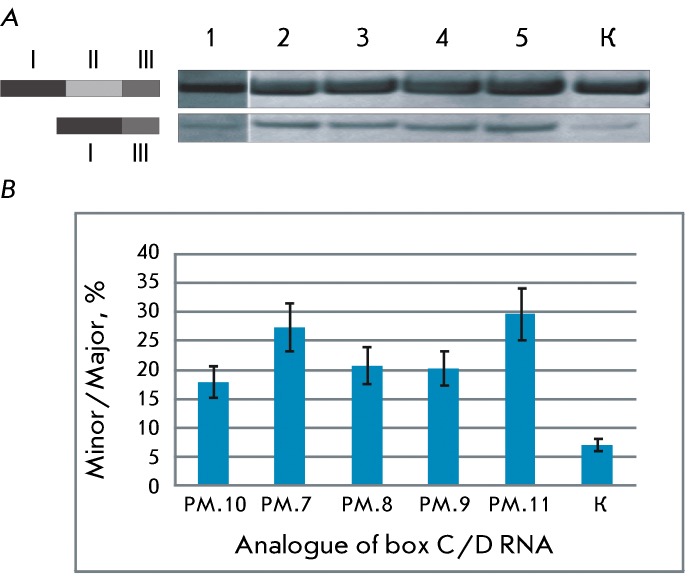
Influence of U24 RNA analogues on pre-mRNA *HSPA8* splicing. ( *A* ) – RT-PCR products of pre-mRNA *HSPA8* splicing variants. The lanes correspond to the cDNA amplification products of 1 – 5 cells transfected with analogues PM.10, PM.7, PM.8, PM.9, PM.11, respectively; K – control MCF-7 cells incubated with lipofectamine. PCR products were analyzed on a 4% native polyacrylamide gel. Pre-mRNA *HSPA8* splicing variants are schematically represented. ( *B* ) – the ratio between the yields of the PCR products of the minor and major pre-mRNA hsc70 splicing variants. РМ.7 – РМ.11 – the cells were transfected with box C/D RNA analogues РМ.7 – РМ.11, respectively. K – control MCF-7 cells were incubated with lipofectamine alone.

**Fig. 3:**
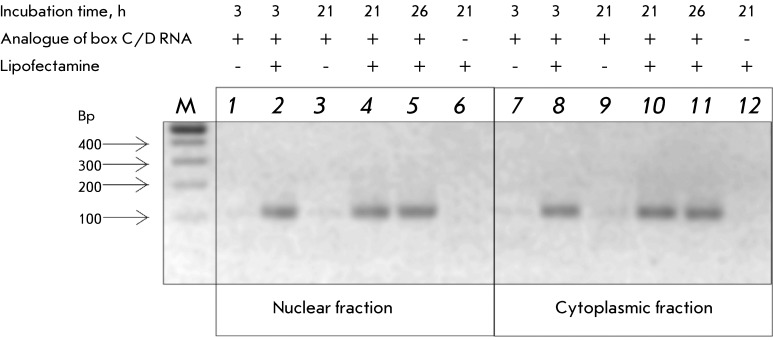
Integrity of artificial box C/D RNA within MCF-7 cells. Human cells were transfected with the PM.8./lipofectamine complex ( *2, 4, 5, 8, 10, 11* ), or PM.8 without lipofectamine ( *1, 3, 7, 9* ) for the time indicated. Control cells were incubated with lipofectamine alone ( *6, 12* ). RT-PCR products of RNA isolated from the nuclear ( *1–6* ) or cytoplasmic fraction ( *7–12* ). DNAs were analyzed on a 2% agarose gel. M – DNA molecular weight marker.

The transfection efficiency, distribution, and stability of the synthetic analogues of box C/D RNAs were assessed via RT-PCR of nuclear and cytoplasmic RNA of the MCF-7 cells transfected with PM.8 RNA. It is clear from the data shown in *Fig. 3* that, 3 * * h after the passive transfection of the cells with PM.8 RNA in the absence of lipofectamine, RNA could be detected neither in the nuclear nor in the cytoplasmic fraction of MCF-7 cells ( *Fig* . *3, lanes 1, 7). * After the cells were transfected with the analogue of box C/D RNA in the presence of lipofectamine, PM.8 RNA was found both in the cytoplasmic and nuclear fractions even 26 h post-transfection ( *Fig* . * 3, lanes 5, 11). * All these facts lead one to the conclusion that synthetic box C/D RNAs in the presence of lipofectamine can efficiently penetrate into the cell cytoplasm and nucleus, where they can participate in the processing of the pre-mRNA-target. 

The RT-PCR data are in close correlation with the results of the analysis of the distribution of FAM-labelled PM.8 RNA in MCF-7 cells via fluorescent microscopy. Thus, after the cells are incubated in a medium with FAM-labelled PM.8 box C/D RNA in complex with lipofectamine, RNA is captured, internalized, and then distributed both over the cytoplasm and the cell nucleus ( *Fig* . *4* ).

**Fig. 4:**
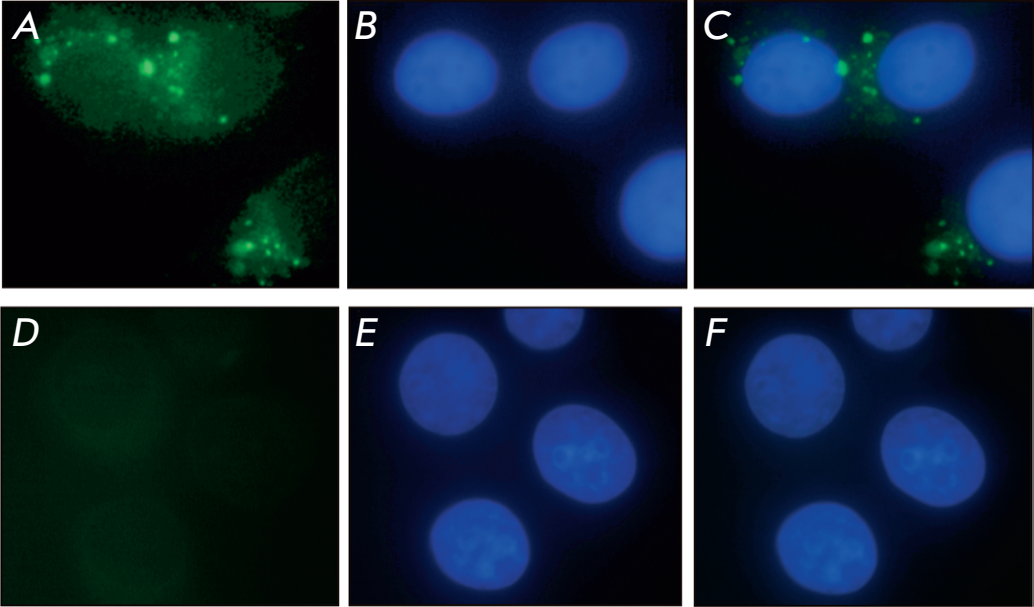
Accumulation of box C/D RNA analogue PM.8 within MCF-7 cells. Fluorescence microscopy images of the transfected ( *A–C* ) and control cells ( *D–F* ): ( *A, D* ) – green filter ( FAM-labelled box C/D RNA analogue); ( *B, E* ) – blue filter (staining with DAPI); ( *C, F* ) – merged images.

It is known that rRNA nucleotides, which directly participate in the formation of ribosomal active sites or are located in close proximity to them, are more likely to undergo post-transcriptional modifications ( pseudouridylation and 2’-O-methylation) [11]. These data permit the assumption that the post-transcriptional modifications have a substantial effect on the rRNA structure during the assembly process and eventually determine the ribosomal functionality [11–13].

**Fig. 5:**
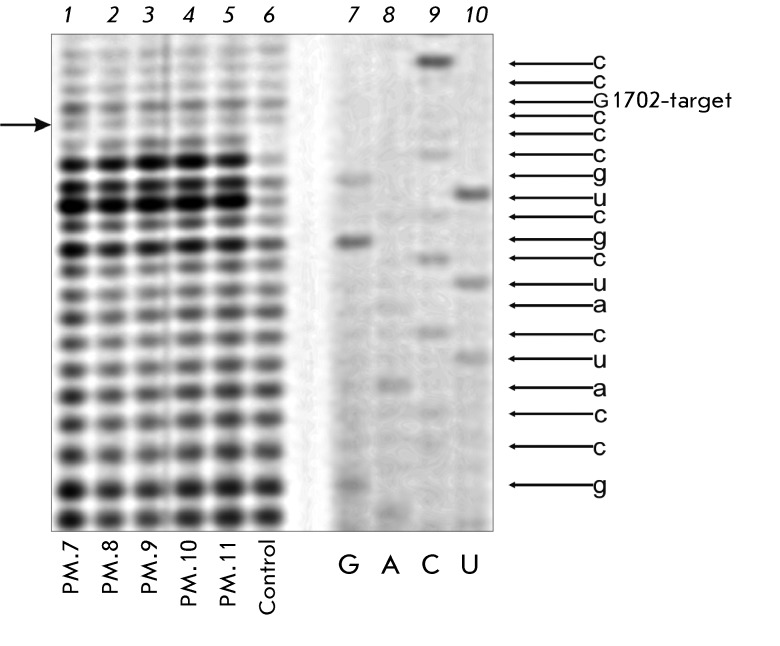
Analysis of 2’-O-methylation of G1702 in 18S rRNA. RT products: ( *1–5* ) – rRNA isolated from MCF-7 cells transfected with box C/D RNA analogues PM.7–PM.11, respectively; ( *6* ) – rRNA isolated from control MCF-7 cells; ( *7–10* ) – sequencing of the corresponding region of human 18S rRNA. RT products were separated on a 12% denaturing polyacrylamide gel. The left arrow points to the position of the cDNA product, whose diminishing intensity could be an indicator of the 2’-O-methylation of G1702 in 18S rRNA.

The induction of 2’-O-methylation of the target of the second guide sequence (G1702 of the 18S rRNA) was analyzed in order to assess the ability of an analogue of box C/D RNA to direct rRNA modification. Since G1702 is one of the key nucleotides of the decoding site of human ribosomes, it was selected to be the target [14]. No additional 2’-O-methylated nucleotide at position G17012 of 18S rRNA was detected in the structure of rRNAs from the MCF-7 cells transfected with synthetic analogues via partial alkaline hydrolysis ( *Fig* . * 5* ). The presence of the 2’-O-methylated nucleotide within rRNA results in a decrease in the yield of the cDNA product (among the products of reverse transcription of statistically hydrolyzed rRNA), the length of which is determined by the position occupied by a nucleotide that is downstream adjacent to the 2’-O-methylated nucleotide [8]. However, it is clear from *Fig. 5* that the yield of the cDNA transcript corresponding to the 2’-O-methylated G1702 of 18S rRNA does not decrease as a result of the transfection of human cells with any analogue of box C/D RNAs.

**Table 2:** Synthetic analogues of U24 box C/D RNA directed to the nucleotides in human 18S and 28S rRNA

Notation	Nucleotide sequence*	Target nucleotide in human rRNA
РР.1827	5’-GGGUGCAGAUGAUGUAAAAUAGCGACGGGCGGUGCUGA GAGAUGGUGAUGACCUUGUUACGACUUUCUGAUGCACCC-3’	U1827 18S
РР.4499	5’-GGGUGCAGAUGAUGUAAAAUAGCGACGGGCGGUGCUGA GAGAUGGUGAUGAACGGUCUAAACCCAGCUGAUGCACCC-3’	G4499 28S
РР.4500	5’-GGGUGCAGAUGAUGUAAAAUAGCGACGGGCGGUGCUGA GAGAUGGUGAUGAGACGGUCUAAACCCACUGAUGCACCC-3’	U4500 28S
РР.4502	5’-GGGUGCAGAUGAUGUAAAAUAGCGACGGGCGGUGCUGA GAGAUGGUGAUGAACGACGGUCUAAACCCUGAUGCACCC-3’	U4502 28S

* See note in *Table 1* .

The effect of synthetic analogues of box C/D RNAs on the post-transcriptional modification of rRNAs in human cells was analyzed using the designed and obtained analogues of U24 box C/D RNA, whose first guide sequence was directed to the nucleotides of human 18S and 28S rRNA. The nucleotide sequences of the analogues of box C/D RNAs are listed in *Table 2.* The targets selected are the key nucleotides of the ribosomal functional sites: U1827 of the 18S rRNA can be found in the ribosomal decoding centre; G4499, U4500, and U4502 of the 28S rRNA, in the peptidyl transferase centre [3, 4]. The second guide sequence of the analogues (the D’-box-dependent one) is directed to G1702 of the human 18S rRNA ( *Table 2* ).

**Fig. 6:**
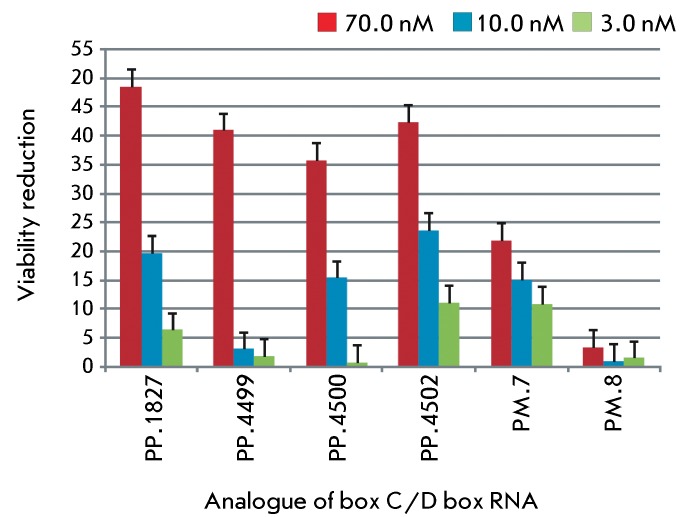
Influence of artificial box С/D RNAs on the viability of MCF-7 cells. The MCF-7 cells were transfected with the RNA/lipofectamine complex and incubated for three days. The data represent the viability reduction (the average decrease in the MTT index and SD in 3 independent experiments) of cells treated with 3.0 nM (green); 10.0 nM (blue); and 70.0 nM (red) of RNA. The relative reduction in viability by 0% corresponds to the MTT index of the cells treated with lipofectamine alone.

A comparative analysis of the effect of the analogues of box C/D RNAs on MCF-7 cell viability was performed. The effect of the analogues of box C/D RNA was assessed after the cells were incubated in a medium with initial RNA concentrations of 3.0, 10.0, and 70.0 nM. It is clear from *Fig. 6* that the greatest reduction in viability (>** 35%) was induced by box C/D RNAs directed to U1827 of the 18S rRNA, G4499, U4500, and U4502 of the 28S rRNA. Meanwhile, the decrease in cell viability and proliferation caused by the analogues of U24 RNA, whose first guide sequence was directed to pre-mRNA of the hsc70 protein, was less than 22% ( *Fig* .  *6, РМ.7 and РМ.8* ).

MCF-7 cells were transfected with the analogues of box C/D RNA ( *Table 2* ) in order to analyze the induction of *de novo * 2’-O-methylation of target nucleotides; 2’-O-methylated rRNA nucleotides were detected by the partial alkaline hydrolysis method. It was ascertained that the contribution of rRNA forms containing the 2’-O-methylated target nucleotide in the total RNA pool of the transfected MCF-7 cells was lower than the sensitivity threshold of the detection technique.

The data relating to the changes in cell viability under the action of box C/D RNA, combined with the observed absence of the modification of the target nucleotides, allows one to assume that the effect of the analogues on human cells can be stipulated by the participation of box C/D RNAs not only in 2’-O methylation of rRNA nucleotides, but in other stages of post-transcriptional rRNA processing and ribosomal assembly, as well.

## DISCUSSION

The conserved structural elements of box C/D RNA, namely, C/C’ (RUGAUGA) and D/D’ (CUGA) boxes, and the guide sequence ensure the ability of these RNAs to participate in the formation of a catalytic complex with proteins of the methyltransferase complex and direct 2’-O-methylation of a pre-specified nucleotide [1, 2, 15]. The guide sequence region of box C/D RNAs is a sequence consisting of 10–21 nucleotides, which is complementary to the RNA target; the nucleotide to be methylated is complementary to the fifth nucleotide of snoRNA upstream from the D box [7].

rRNAs and snRNAs are the major targets of box C/D RNAs in eukaryotic cells. Meanwhile, snoRNAs participating in pre-mRNA processing have been detected [6]. It was ascertained earlier that box C/D RNAs can interact with the transcripts synthesized by RNA polymerase II and guide 2’-O-methylation of a pre-specified nucleotide of the RNA target. The efficiency of the target nucleotide modification is substantially lower than that of the RNA targets synthesized in the nucleus by RNA polymerase I [2]. Moreover, it is a known fact that the chemical modification (in particular, methylation) of the 2’-OH groups of the oligonucleotides participating in pre-mRNA splicing significantly affects the efficiency of pre-mRNA maturation stages [16]. Therefore, the analogues of box C/D RNAs directing the 2’-O-methylation of pre-mRNA nucleotides are a promising model for the design of agents for splicing regulation.

The analogues of U24 small nucleolar box C/D RNA directed to pre-mRNA nucleotides of the *HSPA8 * gene encoding heat shock cognate protein (hsc70) were designed and obtained in the present study ( *Fig. 1* ). It was ascertained that the transfection of human cells with synthetic analogues of box C/D RNA directed to the splice donor and splice acceptor sites of the second intron, adenosine the splicing branch point, the first and the second nucleotides of the first intron of pre-mRNA of the *HSPA8* ( *Table 1* ) results in an increase in the amount of the splicing product of pre-mRNA without the second exon ( *Fig. 2* ). Two key mechanisms can be proposed for the influence of the analogues of box C/D RNA on pre-mRNA target processing: 2’-O-methylation of the target nucleotide and complementary interaction between the antisense region of the box C/D RNA with the target RNA. Both pathways may theoretically result in the inhibition of certain splicing steps and, consequently, in a change in variants of alternative splicing of the pre-mRNA target.

The methods for detection of 2’-O-methylated nucleotides, which are currently in widespread application, do not allow to reveal these modifications in mRNA and pre-mRNA with an adequate level of efficiency. Therefore, it cannot be unambiguously stated whether the methylation of the nucleotide target actually occurs, and whether or not the effect on splicing is caused by 2’-O-methylation of the pre-mRNA target. The key points of pre-mRNA splicing are sensitive to the modifications of the 2’-OH groups of ribose residues to different extents [16]. The resulting data do not permit one to claim considerable differences in the efficiency of splicing suppression by the analogues of box C/D RNA directed to various pre-mRNA nucleotides; therefore, the possibility cannot be excluded that the changes in the ratio between the forms of the alternative splicing of the pre-mRNA target being observed are induced by splicing inhibition by antisense RNA via the mechanism that was described for various oligonucleotide derivatives [17–19].

In order to participate in splicing, artificial RNA has to interact with the pre-mRNA target inside the nucleus. It was demonstrated that the analogues of box C/D RNA in the presence of lipofectamine are capable of efficient penetration into human cells ( *Figs* . * 3, 4* ). It is clear from *Fig* . * 3 * that the analogue of box C/D RNA was reliably detected by RT-PCR 26 h following the single transfection in the presence of lipofectamine in the nuclear and cytoplasmic RNA fractions of MCF-7 cells. The resulting data attest to the fact that the artificial box C/D RNAs are potentially available for interaction with RNA targets located inside the nucleus. The accumulation of synthetic RNA in cells was confirmed via fluorescence microscopy ( *Fig* . * 4* ). Furthermore, it was ascertained that the synthetic analogues of box C/D RNA in the presence of lipofectamine can be efficiently conserved and detected via RT-PCR in human cells 72 h after the single transfection (the data are not illustrated). Meanwhile, in the case of transfection in the absence of lipofectamine, synthetic RNAs cannot be detected in human cells via RT-PCR as early as 3 h after having been introduced into the culture medium ( *Fig* . * 3* ).

For all the analogues of U24 snoRNAs obtained, one of the two guide sequences was designed so as to direct the 2’-O-methyaltion of G1702 in human 18S rRNA ( *Table 1* ). It was shown earlier that the transfection of human cells with synthetic analogues of box C/D RNAs directed to rRNA nucleotides ( *Table 2* ) induces the termination of reverse transcription on the target nucleotides [20, 21]. However, no additional 2’-O-methyl group at the specified position was detected from the analysis of 2’-O-methylation of G1702 in 18S rRNA of the cells transfected with the analogues of box C/D RNA via partial alkaline hydrolysis ( *Fig* . *5* ). Neither of the *de novo* 2’-O-methylation of rRNA nucleotides – the targets of the analogues specified in *Table 2* (after cell transfection with the corresponding RNAs) – was revealed. Meanwhile, the positions of a number of known 2’-O-methylated nucleotides in human rRNA was successfully determined using this method.

The following data are to be given additional consideration in order to interpret the absence of a modification of the target nucleotides (with an exception for the known limitations of the analysis methods [8]). It is a known fact that the participation of box C/D RNA in the 2’-O-methylation of human cell rRNA is possible only provided that this RNA can be recognized by the proteins that are the subunits of the methyltransferase complex (namely, fibrillarin, NOP56p, NOP58p, and 15.5 kDa) and that the complex is formed with the participation of box C/D RNAs [22–24]. Therefore, the low yield of the targeted 2’-O-methylation observed can be explained by the low efficiency of the assembly of catalytically competent methyltransferase complexes with the analogues of box C/D RNA. Moreover, it was previously shown by *Liu B. et al.* [3, 4, 25] (who studied the transfection of yeast cells with DNA constructs encoding the analogues of box C/D RNA) that expression and maturation of artificial snoRNAs take place in the transfected cells. It also turned out that the expression of box C/D RNAs directed to a number of rRNA nucleotides resulted in a significant decrease in the proliferation rate and viability of the cells. The combination of the resulting data made it possible to arrive at a conclusion that it is 2’-O-methylation of rRNA nucleotides, guided by the analogues of box C/D RNA, that is the major reason for the influence of DNA constructs on the proliferation rate of the cells. However, for a number of box C/D RNAs [3, 4, 25], only a low level of 2’-O-methylation (or no modification at all) in the target nucleotides was revealed. On the other hand, the post-translational modifications (2’-O-methylation and pseudouridilation) are known to occur at the stage of maturation of the 47S rRNA precursor [7, 26–28]. The quality of the rRNA transcript is verified at the stage of the assembly of functional ribosomes; the incorrect nonfunctional RNA transcripts undergo degradation in exosomes [29]. The synthetic analogues of box C/D RNA are directed to the rRNA nucleotides immediately participating in the formation and functioning of the ribosomal active sites. Presumably, 2’-O-methylation of rRNA, guided by synthetic analogues of box C/D RNAs, has a significant effect on the rRNA structure and ribosomal functionality; this results in a rapid degradation of modified RNA and its low content in the transfected cells [25, 29].

Despite the fact that no modification of the target nucleotide was observed, artificial box C/D RNAs may complementarily interact with the rRNA target, thus participating in the regulation of the maturation of rRNA transcripts and in ribosomal assembly and functioning. Hence, synthetic analogues of box C/D RNA can participate in the vital activity of the transfected cells and, therefore, affect the viability and proliferation of human cells.

The effect of the analogues of box C/D RNA on the viability of human MCF-7 cells was assessed via the MTT assay. The data obtained made it possible to arrive at the conclusion that cell transfection with synthetic analogues, whose first guide sequence is directed to the nucleotides of 18S and 28S rRNA ( *Table 2* ), results in a decrease in their viability by 36–48%; the initial concentration of synthetic RNA in the medium being 70.0 nM ( *Fig* . * 6, РР.1827, РР.4499, РР.4500, РР.4502* ). Meanwhile, synthetic analogues simultaneously directed to the pre-mRNA of heat shock cognate protein hsc70 and on G1702 in 18S rRNA ( *Table 1* ) reduced the cell’s viability by only 20–25% within an initial concentration range of 3.0–70.0 nM ( *Fig. 6, РМ.7, РМ.8* ).

The rates of proliferation and monolayer formation decreased considerably, and cell morphology changed after the MCF-7 cells were transfected with synthetic analogues of box C/D RNA directed to U1827 in 18S rRNA, G4499, U4500, and U4502 in 28S rRNA. The rate of the decrease in viability varied for different analogues and depended on the target nucleotide and the initial RNA concentration in the culture medium ( *Fig. 6* ).

The difference in the influence of synthetic analogues of box C/D RNA on MCF-7 cell viability attests to the fact that these RNAs are involved in the regulation of vital processes in human cells during the transfection. The fact that this difference is a result of changes in the structure of the guide sequence allows one to assume that post-transcriptional processing of pre-rRNA is the major process modulated by the analogues of box C/D RNA. It should be noted that rRNA nucleotides comprising the ribosomal active sites (the decoding and peptidyl transferase ones) were selected as targets ( *Table 2* ). It was shown earlier by *Liu B. et al.* [3, 4, 25] that the expression of box C/D RNAs directed to these nucleotides in yeast cells induces the suppression of their growth and results in the partial degradation of rRNA.

The suppression of human cell viability induced by the analogues of box C/D RNA attests to the fact that they participate in the regulation of the viability process of the transfected cells. The fact that no target nucleotide modification occurred allows one to put forward a hypothesis that the analogues of box C/D RNA directed to rRNA are weakly involved into the 2’-O-methylation of rRNA nucleotides; however, there is a presumption that they participate in the other stages of post-transcriptional rRNA processing and in ribosome maturation.

## CONCLUSIONS

It has been shown in this study that the transfection of MCF-7 cells (human breast adenocarcinoma cells) with synthetic analogues of box C/D RNA directed to pre-mRNA nucleotides of heat shock cognate protein hsc70 results in the disruption of splicing of the pre-mRNA target. The transfection of MCF-7 cells with analogues of box C/D RNA directed to the nucleotides in 18S and 28S rRNA that play the key role in ribosome functioning induces a decrease in cell viability. The data obtained attest to the high potential of designing constructs for the regulation of human gene expression and translation based on the snoRNA structure. 

## Abbreviations

rRNAs: ribosomal RNAs
snoRNAs: small nucleolar RNAs
snRNAs: small nuclear RNAs
RT-PCR: reverse transcription polymerase chain reaction
RP-HPLC: reverse phase high-performance liquid chromatography
MTT: 3-(4,5-Dimethylthiazol-2-yl)-2,5-diphenyltetrazolium bromide
FAM: carboxyfluorescein
